# Photoacoustic imaging of cells in a three-dimensional microenvironment

**DOI:** 10.1186/s12929-019-0594-x

**Published:** 2020-01-17

**Authors:** Wei-Wen Liu, Pai-Chi Li

**Affiliations:** 1 Graduate Institute of Biomedical Electronics and Bioinformatics, National Taiwan University, Taipei, Taiwan; 2 Department of Electrical Engineering, National Taiwan University, Taipei, Taiwan

**Keywords:** Photoacoustic imaging, Biomedical imaging, Three-dimensional cell culture, Tumor microenvironment

## Abstract

Imaging live cells in a three-dimensional (3D) culture system yields more accurate information and spatial visualization of the interplay of cells and the surrounding matrix components compared to using a two-dimensional (2D) cell culture system. However, the thickness of 3D cultures results in a high degree of scattering that makes it difficult for the light to penetrate deeply to allow clear optical imaging. Photoacoustic (PA) imaging is a powerful imaging modality that relies on a PA effect generated when light is absorbed by exogenous contrast agents or endogenous molecules in a medium. It combines a high optical contrast with a high acoustic spatiotemporal resolution, allowing the noninvasive visualization of 3D cellular scaffolds at considerable depths with a high resolution and no image distortion. Moreover, advances in targeted contrast agents have also made PA imaging capable of molecular and cellular characterization for use in preclinical personalized diagnostics or PA imaging-guided therapeutics. Here we review the applications and challenges of PA imaging in a 3D cellular microenvironment. Potential future developments of PA imaging in preclinical applications are also discussed.

## Introduction

In the past few decades, the conventional 2D cell cultures have remarkably increased the knowledge in basic cell biology and preclinical biomedical applications. However, cells cultured in a 2D monolayer lack a typical 3D architecture. Moreover, cells inhabiting a rigid surface without a 3D elastic fibrous meshwork (i.e., the extracellular matrix [ECM]) cannot normally response the physical or biochemical cues from the surrounding physiological matrix substrate [1–5]. Recently, in efforts to target the tumor microenvironment for improving both the effectiveness and efficiency of cancer therapeutics, several studies such as immunotherapy, tumor vasculature, or ECM remodeling has been led to a new era and yielded novel insights [6–8]. For example, extravasated T lymphocytes infiltrated into the stromal ECM for migrating to the targeted tumor sites has been demonstrated in 3D cell culture models [9–11]. An amoeboid shape change and contact guidance during T-cell locomotion in 3D collagen fibrils has been documented as a protease-independent process, but it should be dependent on protease in a dense collagen fibrillar meshwork with size-limiting pores [9, 10, 12–15]. A similar model for tumor cell migration in the stromal ECM has also been described [13, 15]. Tumor cell intravasation and extravasation through the vascular wall to other organs is a critical step of tumor migration and metastasis [16]. 3D cell cultures have been utilized in biomimetic models of the tumor vasculature or angiogenesis for investigating the intrinsic or extrinsic modulation of the tumor vascular niche [17–20]. Preclinical studies of the normalization of tumor vasculature or drug screening for anti-angiogenesis have also been performed [21, 22]. 3D culture models can recapitulate partial physiological diversity and thereby allow for dissecting underlying regulatory mechanisms into separate units within a controllable microenvironment. The examinations performed in these studies may help to improve therapeutic interventions and inform clinical decisions.

The availability of appropriate imaging modalities for analyzing cell dynamics within 3D cell culture systems can facilitate interpretations and precise quantification. High-resolution imaging systems such as laser-scanning confocal microscopy, electron microscopy, and multiphoton microscopy are usually employed by researchers to quantify and track cell behaviors. However, 3D cell cultures are usually thick and exhibit strong light scattering, which results in the impinging light experiencing severe diffraction and diffusion. One method for acquiring images of high quality during live cell imaging is to culture cells on the surface of a thin layer of 3D ECM matrix gel (also named a 2.5D culture) or a cell-laden 3D ECM matrix gel with a reduced thickness. Microscope objective lenses with numerical apertures (NA) necessary for high-resolution imaging have very small working distances, such as 100–200 μm for lenses with magnifications above 60×. When using an inverted microscope, the thickness of the holder (e.g., coverslip or polydimethylsiloxane layer) supporting the 3D cell culture will reduce the working distance to 0–50 μm, and so the deepest visible site of the 3D gel will be only 50 μm from the bottom surface of the holder. However, when the thickness of a hydrogel is less than 50 μm, a finite-element model simulated that the hydrogel stress field around the indenter actually interacted with the rigid bottom support, leading to a stiffer response [23]. Experimental evidence further proves that the cell aspect ratio, area, and migration speed are significantly increased in hydrogel with a height of less than 200 μm due to the mechanical gradient along its height [23]. Moreover, although yes-associated protein (YAP) has been documented as a key factor to mediate cancer progression through mechanotransduction, a recent report challenges the established knowledge that the breast cancer progression is regulated by YAP-dependent mechanotransduction in 2.5D culture model, which is, the independency of YAP in ECM stiffness-mediated breast cancer progression is found in 3D cultures and patient samples [24]. Therefore, data obtained from 2.5D cultures and thin cell-laden 3D cultures should be examined carefully. Obtaining detailed information about the center region of 3D cell cultures usually requires the biochemical processing of gel fixation following by thin sections of embedded gels to produce samples whose structural, histological, or protein expression patterns can be investigated using optical imaging systems. Regrettably, these processing methods can cause gel deformation or damage, the loss of localized enzymes and metabolite profiles, and alterations to cell dynamics and chemical and nutrient gradients.

To address the problem of deep imaging, PA imaging as a noninvasive and hybrid imaging modality that combines optical excitation and ultrasonic detection to attain better spatial resolution than traditional ultrasound (US) imaging and also achieve deeper penetration than purely optical imaging systems. PA imaging is a powerful imaging technique that can provide scalable and multicontrast images of 3D cell culture scaffolds, ranging from single cells to an organoid culture. Furthermore, both structural and functional information can be obtained using a single- or multiwavelength laser. Conventional optical imaging using contrast agents with emitted fluorescence or bioluminescence, which typically can be imaged with spatial resolution and the imaging depth in micrometer or sub-micrometer scale. By taking the advantage of the laser-based PA principles, photons can be converted into ultrasonic waves in biological samples. Because of acoustic wave can travel through scattering tissue much far than photon does, PA imaging techniques can surpass the depth limitation of optical imaging systems. To provide a practical guide for choosing the appropriate technologies to examine the 3D structural or functional information of biomaterials, cellular behaviors, and cell–biomaterial interactions, we compare the properties of the most widely used imaging modalities to that of PA imaging modality (Table 1). As such, we summarized the scalability, the chemical sensitivity, and the potential applications of acoustic imaging, optical imaging, PA imaging, and electron imaging. Among these techniques, PA imaging can achieve better spatial resolution than acoustic imaging, and its imaging depth can be larger than optical imaging and electron imaging. In this review, we first briefly outline the importance of using 3D cell cultures as novel physiological mimicry platforms, and then discuss the current challenges in optic-based imaging of 3D cell cultures for the characterization of cell–biomaterial interactions. Since PA imaging can potentially obtain images at greater depths, we describe the physical background into how PA imaging works and the principles of the two main PA imaging modalities. Combining PA imaging with the use of multiplex contrast agents makes it possible to monitor interactions between cells and 3D scaffolds. Since most 3D cell cultures have no endogenous contrast agents, the application of exogenous contrast agents in 3D cell cultures will be more focused in this review. Finally, we draw conclusions about the current bottlenecks and the future outlook on expanding the capabilities of PA imaging through the use of multimodality and unconventional imaging toolkits.

**Table 1:** Comparison of properties of the imaging modalities^a^

Imaging modality	Spatial resolution	Imaging depth	Chemical sensitivity	Potential applications
EM	1 nm	0.1 μm	N.A.	Biomineralization, biomaterial surface structure, pore formation, cell–biomaterial interaction
CM	0.4 μm	0.1 mm	High	Cell behavior, cell tracking, gene expression, cellular substructure, functional activity
MPM	1 μm	1 mm	High	Biomaterial architecture, cell tracking, functional activity
OR-PAM	2.5 μm	1 mm	High	Cell tracking, functional activity, fluid dynamics, cell–biomaterial interaction
OCT	10 μm	2 mm	Low	Vascularization, biomaterial architecture, functional activity
AR-PAM	45 μm	3 mm	High	Vascularization, biomaterial architecture, cell metabolism, functional activity
US (20 MHz)	165 μm	3 cm	Low	Fluid dynamics, mechanics, spatial patterning of cells, functional activity

^a^*Abbreviations*: *EM* electron microscopy, *CM* confocal microscopy, *MPM* multi-photon microscopy, *OR-PAM* optical resolution photoacoustic microscopy, *OCT* optical coherence tomography, *AR-PAM* acoustic resolution photoacoustic microscopy, *US* ultrasound imaging, *N.A*. not available

## Review

### Fundamentals of PA imaging

**Fig. 1:**
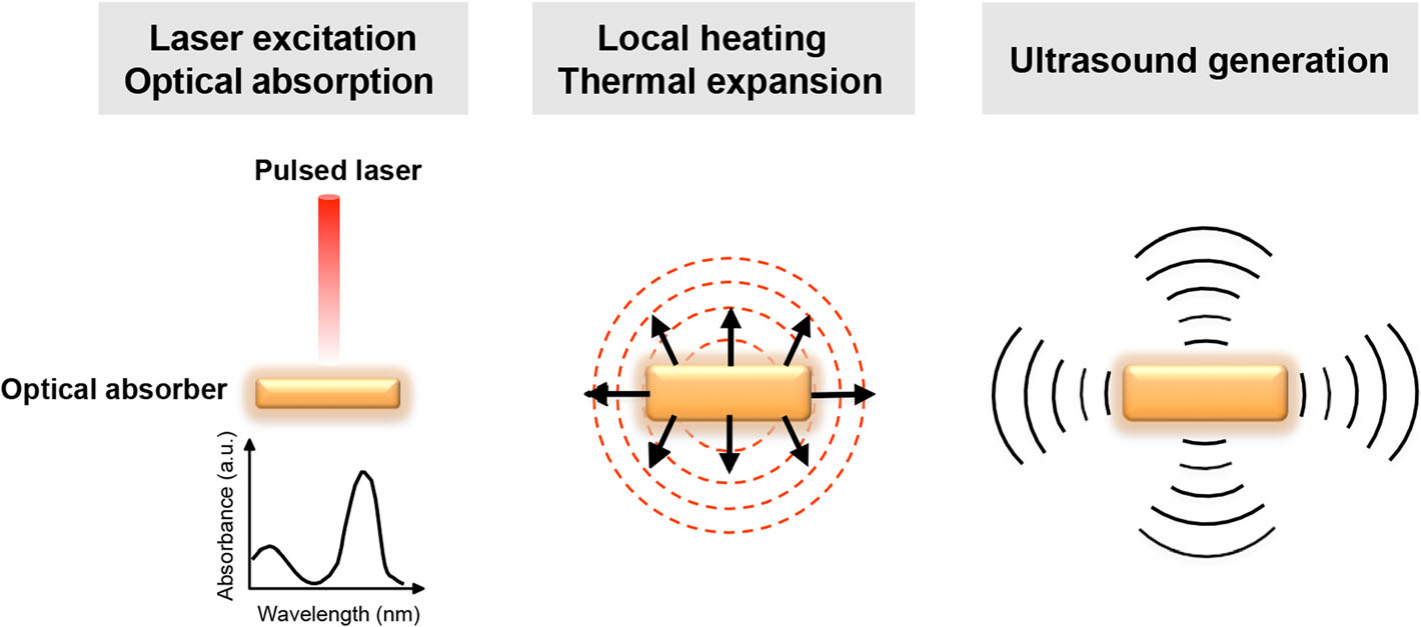
Illustration of PA signal generation. Optical energy excited from a short-pulse laser is absorbed by optical absorbers, which causes an increase in the local temperature. An US pressure wave, the so-called PA signal, is then generated by the thermal expansion of the absorber

PA imaging is based on the physical integration of optical irradiation and ultrasonic detection (Fig. 1) [25–27]. Irradiating light-absorbing materials with a short-pulse laser induces an increase in pressure through thermoelastic expansion. The resulting pressure waves can be interpreted to US waves as the pressure wavefront propagates in the light-absorbing region. The US waves, also known as PA waves, can be detected by US transducers to produce electrical signals. These signals are then amplified, digitized, decoded, and transferred to a computer for image formation. The amplitude of the PA response is proportional to the concentration of the absorbers, the optical absorption coefficient of the photoabsorber, and the thermal coefficient of volume expansion. The contrast of PA imaging when imaging in vivo or in vitro samples can be improved by utilizing the various available PA contrast agents as photoabsorbers such as hemoglobin and gold nanoparticles [28–33].

### Photoacoustic microscopy

Photoacoustic microscopy (PAM) is one type of PA imaging modality that aims to image at millimeter-scale depths and micrometer-scale resolutions. Its microscopic essence is appropriate for visualizing structural, functional, or molecular information such as property alterations of a scaffold, cellular dynamics, or engineered vasculature and angiogenesis inside 3D-scaffold-based samples. During PAM scanning, each laser-pulse-generated time-resolved PA signal recorded from the US transducer is converted into one-dimensional depth-resolved image (A-line) based on the sound velocity in the sample, and A-line scanning is used to form a 2D frame. Coupling this with 2D raster scanning along the horizontal plane allows a 3D image with volumetric information to be generated. Because the degree of scattering is much lower for US than for visible light in biological samples, PAM provides a better spatial resolution and a deeper penetration depth [34, 35].

**Fig. 2:**
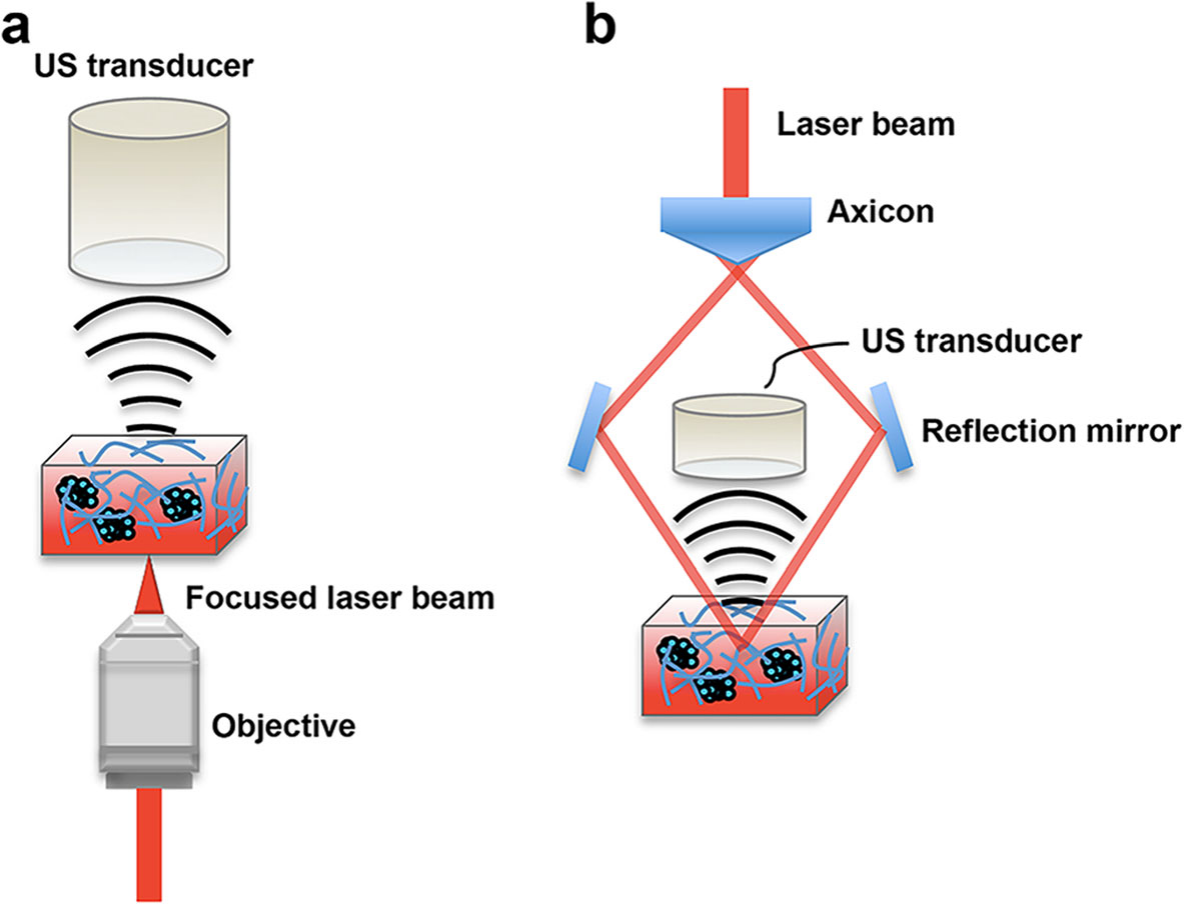
Schematics of two types of PAM system: (a) OR-PAM and (b) AR-PAM. In this setup, 3D tumor spheres labeled with contrast agents are cultured in a cuboidal matrix hydrogel for PA imaging. Note that the laser light is focused in OR-PAM but unfocused in AR-PAM, respectively. Once the laser energy is delivered into the 3D cell culture and absorbed by endogenous or exogenous contrast agents, the absorbed energy is converted into heat, leading to thermal expansion. Ultrasound signals are then generated and detected by the transducer located at the top of the samples

The axial resolution and the achievable penetration depth of PAM are determined by the central frequency of the US transducer. The axial resolution is equal to half the spatial pulse width, and a higher operating frequency has a smaller wavelength and hence generates shorter pulses, giving a better axial resolution. The lateral resolution of PAM is determined by the combined response of the point source from overlapping optical excitation and acoustic detection by the PAM imaging system, known as the point spread function. Depending on what directs the resolution of the imaging system, PAM can be further categorized into optical-resolution PAM (OR-PAM) and acoustic-resolution PAM (AR-PAM) (Fig. 2). In OR-PAM, the optical focus is better than the acoustic focus and a lateral resolution of a few micrometers can be achieved, allowing for single-cell imaging. Nonetheless, high optical scattering limits the penetration depth to around 1 mm in OR-PAM. In AR-PAM, the acoustic focus is much better than the optical focus, and a lateral resolution of a few tens of micrometers can be achieved. The relatively weak acoustic scattering in AR-PAM allows a penetration depth of up to a few centimeters, which enables investigations of phenotypic characteristics in a 3D configuration. In both OR-PAM and AR-PAM, using objectives with low NA makes it possible to image a large field of view without sacrificing the depth resolution.

### Monitoring a 3D microenvironment using PA contrast agents and multiscale PAM

PA contrast agents can be categorized into two types, endogenous and exogenous. Two well-known endogenous contrast agents applied in in vivo label-free PA imaging are melanin and hemoglobin. Except for cells containing melanin, PA contrast is usually undetectable in biomaterials and the cell-laden 3D culture, and so an exogenous contrast agent needs to be introduced for contrast enhancement. Exogenous contrast agents for use in PA molecular imaging have to possess certain photophysical and biological properties, such as efficient optical-to-PA conversion, long-lived excited-state lifetime, biocompatibility, distinct optical absorption spectra (where the endogenous contrast agents have a lower absorption), and the capability to pass through cellular and fibrillar barriers for successful labeling [29, 36]. Furthermore, both endogenous and exogenous contrast agents are usually with optical absorption spectra in the near-infrared (NIR) window (600–1100 nm) so as to ensure their deeper penetration and hence the required imaging depth.

**Fig. 3:**
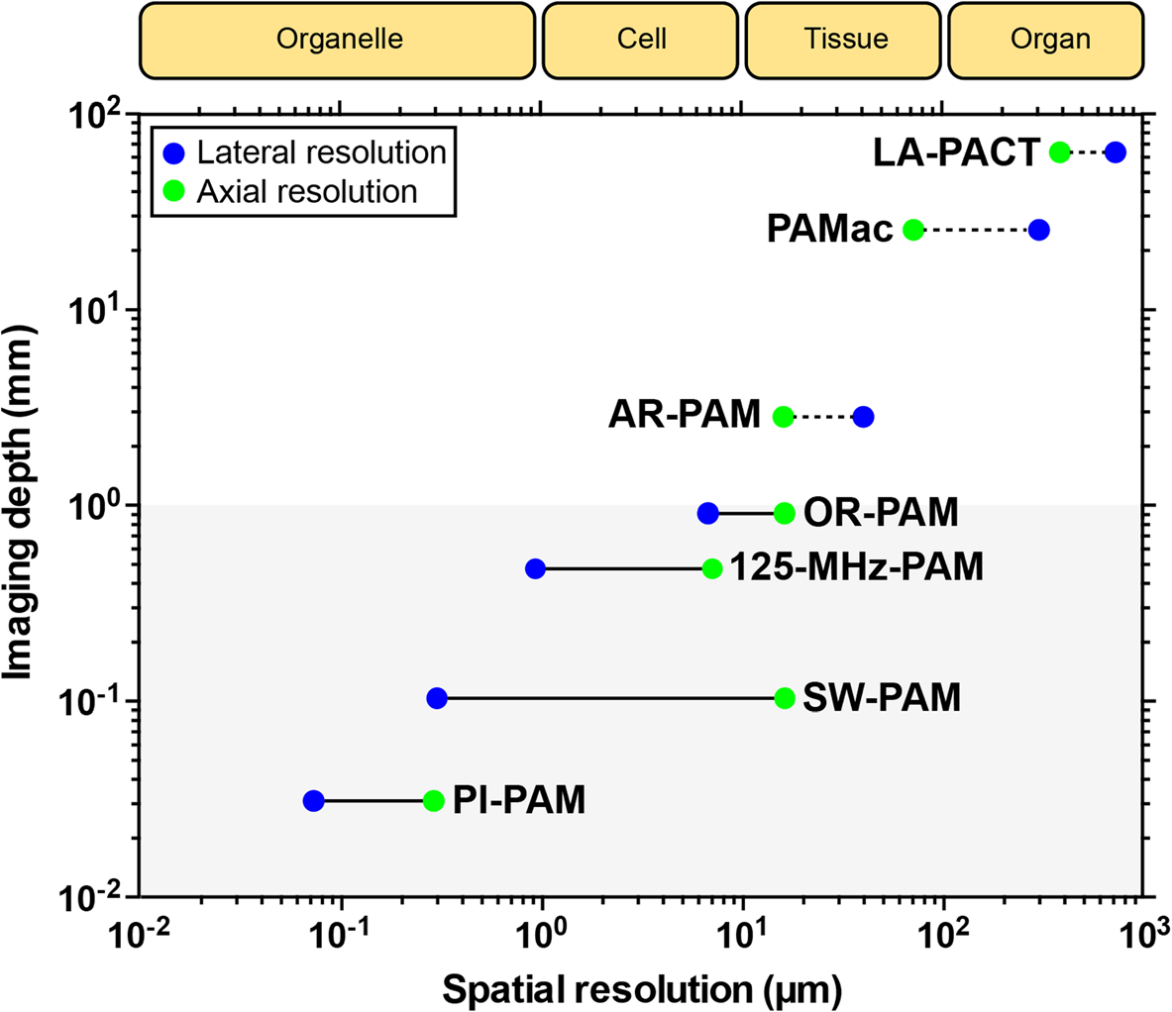
Scalability of PAM among multiscale biological systems. The blue circles denote lateral resolution, and green circles denote axial resolution. Solid lines denote OR-PAMs, and dotted lines denote AR-PAMs. LA-PACT, linear-array PA computed tomography [37]; PAMac, PA macroscopy [38]; AR-PAM, acoustic resolution PAM [39]; OR-PAM, optical resolution PAM [40]; 125-MHz-PAM, PAM using a 125-MHz ultrasound detector [41]; SW-PAM, subwavelength resolution PAM [42]; PI-PAM, photoimprint PAM [43]. Figure adapted from [44]

For multiscale biological systems, several kinds of representative PAM systems are summarized based on the scalable imaging performance shown in Fig. 3 [44]. Generally, AR-PAM (i.e. using unfocused laser beam) can achieve imaging depth beyond 1 mm, in contrast, OR-PAM can only achieve imaging depth within 1 mm due to the limited penetration of a focused laser beam. AR-PAM with a focused 50-MHz ultrasound detector can provide lateral resolution of 45 μm and axial resolution of 15 μm for detection of oxygen saturation in a single blood vessel over 1 mm beneath the tissue surface [39]. The imaging depth can be extended to 4 cm and the lateral resolution is enlarged to 100–560 μm when reducing the center frequency of the focused ultrasound detector to 5 MHz for macroscopic purpose [38]. Real-time imaging and the deeper penetration depth up to 7 cm can be achieved when using an ultrasound transducer array as the detector combined with a computed tomography scanning system [37, 45]. OR-PAM for imaging cells has the lateral resolution of 1–5 μm, and the axial resolution can achieve to ~ 15 μm when combination with a 75-MHz focused ultrasound detector [40] and it can be improved to 7.5 μm when using focused ultrasonic detector with a center frequency of 125 MHz [41]. Combination with objectives with a higher NA and sub-diffraction techniques, the lateral resolution of OR-PAM can be increased to 87 to 220 nm to achieve the purpose for imaging organelle [42, 43]. Following sections will draw on the biomedical applications of PA imaging based on the properties of PA contrast agents including probing functional biological processes, structural imaging of biomaterial scaffolds and vasculature, cell tracking, and tumor detection in 3D microenvironments. Among these studies, to achieve PA imaging at the single-cell scale, OR-PAM can be used, and AR-PAM can be used to achieve deeper penetration and tissue-scale imaging in in vivo animals/human studies.

#### Functional imaging of 3D cell cultures/tissues

The change in the absorption spectra between oxyhemoglobin and deoxyhemoglobin enabled the total concentration of hemoglobin and the oxygen saturation in the rodent brain vasculature or tumor angiogenesis to be detected in multiwavelength PAM [37, 45–47]. Gold nanoparticles (AuNPs) are exemplar PA contrast agents that provide unique possibilities for both in vitro and in vivo molecular PA imaging. For example, AuNPs have been administered to blood vessels for blood flow velocity measurements in chicken breast tissue [48, 49] and for monitoring the intravascular fluid pathway of the rat brain [50]. The use of NIR-responsive PA dyes for functional PA calcium imaging in in vitro 3D cell cultures and in vivo animal models, including arsenazo III, chlorophosphonazo III, and genetically encoded calcium indicators, has also been documented [51–53]. For in vivo or ex vivo functional imaging or to observe flow dynamics in a fluid system, working with AR-PAM can achieve deeper imaging up to few millimeters and spatial resolution of 45–200 μm, but OR-PAM can provide cellular level information such as intracellular calcium imaging in 3D cell culture systems.

#### Structural imaging of 3D scaffolds/tissues

After implanting 3D engineered porous scaffolds into mice ears, neovascularization in the implanted scaffolds could be noninvasively monitored and quantified using both AR-PAM and OR-PAM for up to 6 weeks [54]. Polymer porous 3D scaffolds incorporating carbon nanotubes or 3D-printed alginate-polydopamine scaffolds can be used for structural examinations of the 3D scaffolds with PA imaging [55, 56]. In these studies, to visualize the network of capillaries (5–10 μm) and 3D engineered porous scaffolds, OR-PAM is used to achieve spatial resolution of 5 μm and axial resolution of 15 μm, but with a limited imaging depth (1 mm). OR-PAM provides well-resolved images allowing quantification of the characteristics of the 3D scaffolds such as pore size, porosity, or fiber formation, and AR-PAM provides a thicker image layer up to 2 mm.

#### Cell tracking and tumor cells detection in 3D cell cultures/tissues

Melanin, a naturally produced pigment in melanoma cells, provides good optical and PA contrast in melanoma relative to the surrounding tissue, and allowed for tracing the melanoma cells and monitoring the melanoma growth for 2 weeks [57]. This property means that melanoma cells are traceable for monitoring cell proliferation in engineered 3D porous scaffolds [58]. Making use of cellular endocytosis processes, AuNPs can be loaded into stem cells or macrophages as a PA contrast agent, which opens the possibility of the long-term tracking and monitoring of stem cells or macrophages in a 3D fibrin or gelatin scaffold through multimodal US and PA imaging for utilization in investigations of stem cell therapy [59–65]. Nanoparticles are generally more likely to accumulate in a tumor lesion due to the enhanced permeability and retention of the leaky tumor blood vessels [66], which has been demonstrated by the passive targeting and accumulation of AuNPs at a tumor site [67]. For tracking cells or delivering the contrast agent to specific regions in order to reduce off-target effects, strategies for conjugating the targeting ligands such as antibodies, peptides, and aptamers with contrast agents for active targeting have been developed. AuNPs with molecular targeting ability such as those conjugated with antibodies recognized to tumor protein biomarkers, and Arg-Gly-Asp (RGD) peptide are also commonly applied for tumor detection in vivo in PA imaging [68–73]. Exploring the crosstalk between stromal ECM and T cells is important for the corresponding immunotherapy strategies. T cells that have taken up AuNPs or can be loaded in an in vitro 3D hydrogel for tracking individual T cells when migrating to tumorspheres with OR-PAM [74, 75]. T cells labeled with NIR-797-isothiocyanate (an NIR PA and fluorescent dye) can be applied to imaging the dynamic change of T cells in lymph nodes in an in vivo mouse model by using AR-PAM [76].

One interesting application of using PA exogenous contrast agents is detecting the PA signals of matrix metalloproteinase-2 (MMP-2) in follicular thyroid cancer [77]. MMP-2 is abundant in several kinds of tumor cells and is known to be closely associated with tumor progression and metastasis [78]. MMP-2 can be targeted by a modified activatable cell-penetrating peptide that is labeled with two chromophores exhibiting different optical absorption wavelengths: BHQ-3 (675 nm) and Alexa Fluor 750 (750 nm) [79]. Both chromophores can be detected photoacoustically. Once MMP-2 is cleaved, only the dye with the BHQ3-labeled cell-penetrating part of the probe accumulates in the cells, and the location of the cleaved probe is observable after background subtraction. These synthesized contrast agents were used to noninvasively detect the location of follicular thyroid cancer in a mouse model by using AR-PAM [77] and may be used in 3D tumor culture model as well.

### Bottlenecks and future prospects

To expand the capabilities of multimodality imaging, PAM could be combined with US imaging in image-guided tumor therapies for the purpose of theranostics. The use of both the PAM and US modalities provides anatomical and functional information [32, 80–83]. Contrast agents in multimodality imaging systems can enhance the contrast in two or more modalities. For example, the position of the sentinel lymph node can be displayed using US imaging, with PA imaging used to display the accumulation of methylene blue [83]. Combined PA and US imaging with PA contrast agents can be further applied in image-guided photothermal therapy [52, 71, 72]. An US system could be used to monitor the targeting of AuNPs-encapsulated microbubbles, with PA imaging used to monitor the US-assisted delivery of AuNPs at the tumor lesion [66]. Moreover, phase-shifted droplets can be used as the contrast agent to enhance the contrast of combined US and PA imaging and also the therapeutic effects [28, 84, 85]. These previous studies have mainly relied on an optical droplet vaporization mechanism, and deep explorations of the underlying physics are now required to further optimize these techniques. The potential bioeffects should also be determined to ensure safety. A very recent phantom study employed the cancer drug doxorubicin as a PA contrast agent has shed more light on tumor theranostics [86]. Further phantom and in vitro 3D cell culture validations should be performed to improve these methods with consideration of the tissue complexity before moving to clinical applications.

Another aspect of PA imaging in a 3D cell microenvironment that needs further work is improving the imaging frame rate with the aim of achieving real-time functional applications, especially in thick 3D scaffolds. For example, acoustic-lens-based PA imaging [87, 88] and optical US mapping [89] open up new possibilities to increase the imaging speed, spatial resolution, and field of view. Finally, quantitative studies for standardizing preclinical applications are also important for translating the present results to the clinic.

## Conclusions

PA imaging has been investigated in preclinical studies over the past decade. This review has described the current state of PA imaging, focusing on the application of PA imaging techniques to a 3D cellular microenvironment. PA imaging provides a better penetration depth and can yield both structural and functional information of 3D biological samples from the single-cell level to the organoid level. Combining a multiwavelength laser with the use of contrast agents can produce multicontrast images. Hence, PA imaging has been developed as a powerful tool to dissect the mechanisms underlying spatiotemporal development in preclinical studies. However, it is difficult to compare the results obtained from different 3D cell culture systems and PA imaging systems due to the wide range of the in-house systems that are available. Future works will focus on quantitative studies by using various types of PA imaging systems to achieve standardization of each biological characteristic in different 3D cell culture samples.

## Data Availability

Not applicable.

## Abbreviations

2D: Two-dimensional
3D: Three-dimensional
AR-PAM: Acoustic-resolution microscopy
AuNPs: Gold nanoparticles
CM: confocal microscopy
ECM: Extracellular matrix
EM: electron microscopy
MMP-2: Matrix metalloproteinase-2
MPM: multi-photon microscopy
NIR: Near-infrared
OCT: optical coherence tomography.
OR-PAM: Optical-resolution microscopy
PA: Photoacoustic
RGD peptide: Arg-Gly-Asp peptide PAM Photoacoustic microscopy
US: Ultrasound
YAP: Yes-associated protein
